# Impact of Lactic Acid and Fermented Dairy‐Based Marinades on Quality Attributes and Biogenic Amine Formation in Turkey Breast Meat

**DOI:** 10.1002/fsn3.72185

**Published:** 2026-07-28

**Authors:** Gülsüme Biçakci, Ercan Sarica

**Affiliations:** ^1^ Food Engineering Department, Faculty of Engineering Bolu Abant İzzet Baysal University Bolu Turkey

**Keywords:** acidic marination, biogenic amines, fermented dairy products, physicochemical properties, sensory quality, turkey breast meat

## Abstract

This study investigated the effects of lactic acid (LA), yogurt whey, kefir, and yogurt marinades on the physicochemical properties, sensory quality, and biogenic amine (BA) formation of turkey breast meat. Samples were marinated for 24 h at 4°C and evaluated after marination and subsequent cooking. Fermented dairy‐marinated samples significantly reduced pH compared with the control (*p* < 0.05), with yogurt whey‐marinated samples showing the strongest acidification effect, whereas the lactic acid‐marinated samples did not markedly modify meat pH. Marination significantly influenced color attributes; yogurt increased lightness, and yellowness development after cooking was primarily associated with thermal reactions but remained treatment‐dependent. Marinade absorption differed substantially among treatments, being highest in yogurt‐marinated and LA‐marinated samples and lowest in yogurt whey‐marinated samples, while cooking loss was not significantly affected. Total BA levels exhibited treatment‐dependent numerical variations after marination and cooking, although no significant differences were detected among treatments. Polyamines remained the predominant amines after heat treatment, and histamine levels were low in all samples, indicating no safety concern. Sensory evaluation revealed significant differences only in appearance/color and odor, while kefir‐marinated samples tended to provide improved juiciness and overall acceptability. Overall, fermented dairy‐based marinades enhanced acidity and visual quality without promoting excessive BA accumulation. Yogurt whey, as a dairy by‐product, demonstrated potential as a functional and sustainable marinade for poultry meat processing, contributing to both quality improvement and by‐product valorization.

## Graphical abstract

Turkey breast meat was marinated with lactic acid (pH 4.0), yogurt whey, kefir, or yogurt (1:1, w/w) for 24 h at 4°C and then cooked at 185°C for 15 min. Fermented dairy‐based marinades improved meat quality without promoting excessive biogenic amine formation. Yogurt whey showed the strongest acidification effect, yogurt improved lightness and marinade absorption, whereas kefir exhibited the most favorable sensory performance. These findings demonstrate the potential of yogurt whey as a sustainable and functional marinade alternative for poultry meat processing.

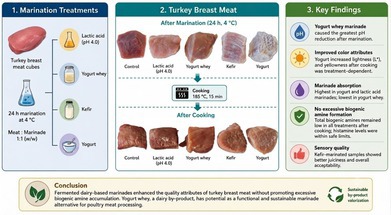

## 1 Introduction

Compared with red meat, poultry meat, especially turkey, is characterized by a high protein content, low fat levels, and a reduced proportion of saturated fatty acids, which contributes to its recognition as a healthier alternative for consumers concerned with cardiovascular health and body weight management (Al‐Baidhani and Al‐Qutaifi 2021). Turkey breast meat is especially preferred because of its lean structure and high protein content; however, these same characteristics often result in technological and sensory challenges, including reduced water‐holding capacity, increased cooking loss, and excessive toughness after cooking (Serdaroğlu et al. 2024). For this reason, technological interventions such as marination are particularly relevant for improving the quality and consumer acceptance of turkey breast meat.

Marination is a widely applied processing technique used to improve meat quality by enhancing tenderness, juiciness, flavor, and microbial stability. Acidic marination with organic acids such as lactic, citric, or acetic acid promotes pH reduction, protein denaturation, connective tissue weakening, and collagen solubilization, thereby improving water‐holding capacity and reducing cooking loss (Aktaş et al. 2003; Ehsanur Rahman et al. 2023). In addition, acidic conditions can inhibit spoilage and pathogenic microorganisms, contributing to improved product safety and shelf life (Yazgan et al. 2021). However, excessive acidification may adversely affect protein integrity, highlighting the need for balanced marination strategies.

In recent years, fermented dairy products and dairy by‐products have attracted increasing attention as natural and functional marination agents. Yogurt, kefir, and whey contain organic acids, milk proteins, bioactive peptides, and fermentation‐derived metabolites that can positively influence meat texture, color, flavor, and microbial stability (Latoch 2020; Latoch and Libera 2019). Yogurt whey is produced in large quantities during the manufacture of strained yogurt and similar fermented dairy products and is often considered an environmental burden due to its high organic load. Its use as a marinade may therefore provide a sustainable strategy for both waste valorization and meat quality improvement (Augustyńska‐Prejsnar, Ormian, Hanus, et al. 2019). Despite differences in their production processes, yogurt whey and acid whey share similar acidification‐related effects that may influence muscle protein structure, water‐holding capacity, and tenderness. Therefore, previous studies on acid whey marination may help explain the potential effects of yogurt whey on meat quality (Sionek et al. 2025; Sokołowicz et al. 2021).

Beyond their acidifying effects, fermented dairy products also provide fermentation‐derived metabolites, commonly referred to as postbiotics, including organic acids, bioactive peptides, and free amino acids. These compounds may influence protein–water interactions, oxidative stability, and microbial metabolism without requiring viable microorganisms, making fermented dairy‐based marinades a mild and clean‐label alternative for meat processing (Gurunathan et al. 2024).

Biogenic amine formation represents an important quality and safety concern in meat products, as these compounds are primarily produced through microbial amino acid decarboxylation and are widely regarded as indicators of microbial activity, product freshness, and hygienic status (Gardini et al. 2016). Processing factors such as marination and cooking can either enhance or limit amine accumulation depending on pH conditions, microbial dynamics, and substrate availability (Latoch et al. 2023; Mozuriene et al. 2016). Consequently, evaluating biogenic amine profiles alongside physicochemical and sensory attributes provides a more comprehensive basis for assessing the overall quality and safety of marinated poultry meat.

Acidic marination may influence meat quality through pH‐mediated modifications in muscle proteins and connective tissue, affecting tenderness, water‐holding capacity, cooking performance, and microbial stability (Aktaş et al. 2003; Moeini et al. 2024). Fermented dairy products such as yogurt, kefir, and whey additionally contain organic acids, lactic acid bacteria, peptides, and other fermentation‐derived metabolites that may contribute to meat quality improvement (Latoch and Libera 2019; Rocha‐Mendoza et al. 2021). In meat systems, microorganisms including Pseudomonas spp., Enterobacteriaceae, lactic acid bacteria, and some Staphylococcus and Micrococcus species may contribute to the formation of biogenic amines through amino acid decarboxylation reactions. Compounds such as putrescine, cadaverine, tyramine, and histamine are frequently associated with microbial activity, protein degradation, and hygienic quality, whereas spermidine and spermine are naturally occurring polyamines commonly present in animal tissues (Bıçakcı and Eren 2026; Gardini et al. 2016). However, information regarding the influence of fermented dairy‐based marinades on biogenic amine formation in poultry meat remains limited.

Therefore, the present study aimed to comparatively evaluate the effects of lactic acid solution and fermented dairy‐based marinades (yogurt whey, kefir, and yogurt) on the physicochemical, sensory, and safety characteristics of turkey breast meat, with special emphasis on biogenic amine formation and the potential of yogurt whey as a sustainable value‐added dairy by‐product for meat marination. It was hypothesized that, despite the presence of fermentation‐derived metabolites and naturally occurring biogenic amines in fermented dairy products, the use of yogurt whey, kefir, and yogurt as marinades would not promote excessive biogenic amine accumulation in turkey breast meat and could provide acceptable physicochemical and sensory quality characteristics compared with a conventional lactic acid solution.

## 2 Materials and Methods

### 2.1 Materials

Fresh turkey breast meat was obtained from a commercial poultry processing plant (Bolca Hindi, Bolu, Turkiye) and transported to the Meat and Meat Products Technology Laboratory of Bolu Abant İzzet Baysal University under refrigerated conditions (4°C ± 1°C) on the same day of production in order to maintain the cold chain. The meat was obtained from bulk commercial production batches as pre‐cut cubes (approximately 2 × 2 × 2 cm; approximately 10 g each), and visible fat and skin were removed prior to use. Food‐grade lactic acid (GRAS status) was purchased from Merck (Germany). Yogurt and kefir were obtained from commercial producers (İçim and Altınkılıç, Turkiye). The yogurt whey used in the experiments was obtained in the laboratory as a by‐product of strained yogurt production by filtering yogurt through a sterile muslin cloth under controlled conditions. The whey was stored at 4°C ± 1°C until use.

### 2.2 Marination Procedure

Marination treatments consisted of a lactic acid solution (LA; adjusted to pH 4.00), yogurt whey, kefir, and yogurt. Cubed turkey breast samples (250 g) were placed in food‐grade polyethylene bags, and marinades were added at a 1:1 (w/w) ratio. Samples were manually mixed for 30 min to ensure uniform penetration of the marinades and subsequently stored at +4°C for 24 h. Control samples were not subjected to any marination treatment.

Prior to marination, the pH and titratable acidity values of the marinade systems were determined. The pH values of the lactic acid solution, yogurt whey, kefir, and yogurt marinades were 3.97, 3.99, 4.45, and 4.14, respectively, while titratable acidity values were 0.0035%, 0.758%, 0.844%, and 1.159%, respectively.

After marination, excess marinade remaining on the sample surfaces was removed by draining the samples on a wire rack for 10 min at room temperature prior to weighing. Marinade absorption was determined based on the percentage weight difference between the initial sample weight and the sample weight after marination and drainage.

Cooking was performed in a preheated electric convection oven (Arçelik Turbo, Bolu, Türkiye) at 185°C for 15 min using baking paper‐lined trays. Following cooking, samples were cooled and subjected to physicochemical, biogenic amine, and sensory analyses. Measurements were conducted before marination, after marination, and after cooking (Bıçakcı et al. 2023).

### 2.3 Physicochemical Analyses

The pH of turkey meat samples was measured by homogenizing 10 g of sample with 100 mL of distilled water using a laboratory blender (Waring Commercial, USA). The pH was recorded using a calibrated pH meter (Schott Instruments Lab 860, UK). Color parameters were determined using a colorimeter (CR‐400; Minolta, Japan) and expressed as L* (lightness), a* (redness), and b* (yellowness) values (Gökalp et al. 2010).

Marinade absorption was determined from the percentage change between the initial sample weight and the sample weight after marination and drainage according to the following equation:

$$\text{Marinade absorption}\;\left(\%\right)=\frac{\text{Weight after marination}-\text{Initial weight}}{\text{Initial weight}}$$

Cooking loss was calculated from the percentage difference between the weight of the drained marinated samples before cooking and the final sample weight after cooking and cooling using the following equation (Bıyıklı et al. 2020):

$$\text{Cooking loss}\;\left(\%\right)=\frac{\text{Weight before cooking}-\text{Weight after cooking}}{\text{Weight before cooking}}$$

### 2.4 Biogenic Amine Analysis

Biogenic amines, including tryptamine (TRP), 2‐phenylethylamine (PhA), putrescine (PUT), cadaverine (CAD), histamine (HIS), tyramine (TYR), spermidine (SPD), and spermine (SPM), were determined according to the method described by Eerola et al. (1993) with HPLC analysis (Flexar, PerkinElmer, USA). Briefly, 2 g of homogenized sample was extracted with 0.4 M perchloric acid containing 1,7‐diaminoheptane as an internal standard and homogenized using an Ultra‐Turrax homogenizer (IKA T18 Digital, Germany). Following centrifugation (3000 rpm, 10 min, +4°C), the supernatant was filtered and subjected to dansyl chloride derivatization.

Chromatographic separation was performed using a C18 column (Aqueous, 250 × 4.6 mm, 5 μm; PerkinElmer, USA) at 40°C with a gradient mobile phase system consisting of acetonitrile and 0.1 M ammonium acetate at a flow rate of 1 mL/min. Detection was carried out at 254 nm using a photodiode array detector (PDA). Standard solutions of TRP, PhA, PUT, CAD, HIS, TYR, SPD, SPM, and 1,7‐diaminoheptane (internal standard) were obtained from Sigma‐Aldrich. Quantification was performed using external calibration curves prepared for each biogenic amine compound.

The analytical procedure used in the present study is a widely established and previously validated HPLC method extensively applied for biogenic amine determination in meat products. Calibration curves showed high linearity (*R* ^2^ > 0.99 for all compounds) (Bıçakcı and Eren 2026).

### 2.5 Sensory Analysis

Sensory evaluation was conducted by a panel of 10 semi‐trained assessors (7 female and 3 male, aged 20–40 years) with normal sensory acuity. The panel consisted of academic staff members and graduate researchers from the Department of Food Engineering, Bolu Abant İzzet Baysal University, and represented a semi‐trained sensory panel familiar with meat product evaluation (Bıçakcı et al. 2023). Sensory sessions were conducted independently for both production batches using the same panelists. Prior to evaluation, panelists were informed about the purpose and procedures of the study, and participation was voluntary. Samples were evaluated for appearance and color, texture (tenderness), juiciness, flavor, odor, and overall acceptability using a nine‐point hedonic scale (1 = dislike extremely; 9 = like extremely), as described by Erge et al. (2018). Cooked samples were equilibrated to room temperature for 10 min, coded with random three‐digit numbers, and served in randomized order.

### 2.6 Statistical Analysis

The study was carried out using two independent production batches performed on different days, and each analytical determination was conducted in duplicate for each batch. Thus, four analytical values were obtained for each parameter.

Data were analyzed using IBM SPSS Statistics 26.0 (SPSS Inc., Chicago, USA). One‐way analysis of variance (ANOVA) was used to determine significant differences among treatments, and mean comparisons were performed using Duncan's multiple range test at a significance level of *p* < 0.05. Considering the limited sample sizes commonly encountered in food and biological experiments, the assumptions required for parametric analyses were considered reasonably satisfied for the obtained datasets, in line with common practices in food science research (Granato et al. 2014).

## 3 Results and Discussion

### 3.1 Physicochemical Properties

The effects of different marination treatments on pH, color parameters (L*, a*, b*), marinade absorption, and cooking loss are presented in Table 1. The initial pH of raw turkey breast meat was 6.40, which falls within the typical range reported for fresh poultry (Al‐Baidhani and Al‐Qutaifi 2021).

**TABLE 1:** pH values, color parameters (L*, a*, b*), marinade absorption, and cooking loss (%) of turkey breast meat marinated with different treatments after marination and after cooking.

Analysis	Raw Turkey meat (Control)	After marination			
		LA	Yogurt Whey	Kefir	Yogurt
pH	6.40 ± 0.04^aA^	6.41 ± 0.04^aA^	5.63 ± 0.03^cA^	5.92 ± 0.12^bA^	5.66 ± 0.01^cA^
L* (lightness)	48.63 ± 1.22^dA^	56.67 ± 1.30^bA^	53.51 ± 0.36^cA^	58.48 ± 0.71^bA^	66.07 ± 0.43^aA^
a* (redness)	16.49 ± 2.12^aA^	11.90 ± 0.33^bB^	13.33 ± 1.40^abA^	12.29 ± 1.37^bA^	6.15 ± 0.06^cB^
b* (yellowness)	4.07 ± 0.70^bB^	5.99 ± 0.76^abB^	7.86 ± 0.37^aB^	5.07 ± 1.14^bB^	4.43 ± 0.45^bB^
Marinade absorption (%)	−0.13 ± 0.00^c^^,^ ^a^	11.54 ± 1.58^a^	0.49 ± 0.10^c^	6.30 ± 0.72^b^	12.16 ± 0.03^a^

Analysis	Control	After cooking			
		LA	Yogurt Whey	Kefir	Yogurt
pH	6.48 ± 0.01^abA^	6.64 ± 0.13^aA^	5.63 ± 0.04^cA^	6.19 ± 0.16^bA^	5.82 ± 0.23^cA^
L* (lightness)	54.34 ± 0.13^bA^	54.42 ± 2.15^bA^	55.43 ± 0.97^bA^	57.42 ± 3.89^abA^	63.03 ± 4.02^aA^
a* (redness)	14.85 ± 1.19^abA^	15.36 ± 0.64^aA^	13.21 ± 0.69^bcA^	12.76 ± 0.35^cA^	9.23 ± 0.16^dA^
b* (yellowness)	15.50 ± 1.12^aA^	16.89 ± 1.51^aA^	14.44 ± 0.16^abA^	15.87 ± 1.16^aA^	12.42 ± 0.75^bA^
Cooking loss (%)	34.25 ± 11.30^a^	42.99 ± 10.90^a^	46.23 ± 11.93^a^	40.72 ± 5.06^a^	44.62 ± 9.28^a^

*Note:* Different lowercase letters indicate significant differences among treatments within the same condition (*p* < 0.05). Different uppercase letters indicate significant differences between conditions within the same treatment (*p < 0.05*). Values sharing the same letter are not significantly different (*p* > 0.05).

Abbreviations: LA, lactic acid; Yogurt Whey, whey obtained from strained yogurt production.

^1^ Negative values indicate moisture loss during 24 h refrigerated storage in control samples without marination.

After marination, significant treatment‐dependent differences in pH were observed (*p* < 0.05). The meat samples marinated with lactic acid solution (6.41) showed pH values comparable to those of raw meat, indicating that the applied lactic acid treatment did not induce a pronounced acidification effect under the experimental conditions. This may be associated with the limited penetration of the acid solution into the muscle structure and the buffering capacity of meat proteins, which can reduce pH changes during short‐term marination. In contrast, fermented dairy‐based treatments resulted in substantially lower pH values. The lowest pH was recorded in the yogurt whey‐marinated samples (5.63), followed by yogurt‐marinated samples (5.66), while kefir‐marinated samples exhibited an intermediate value (5.92). These findings indicate that fermented dairy‐based marinades influenced meat acidification differently from the lactic acid‐marinated samples.

After cooking, pH values increased slightly in both the control and marinated samples compared with the marinated state; however, these changes were not statistically significant within treatments (*p* > 0.05). Thus, heating did not markedly alter the relative acidity established during marination. Nevertheless, the ranking among treatments was preserved: yogurt whey‐marinated samples (5.63) and yogurt‐marinated samples (5.82) maintained the lowest pH values, kefir‐marinated samples remained intermediate (6.19), and the LA‐marinated samples showed the highest pH (6.64). Comparison of marinated and cooked samples indicated that pH values tended to increase in meat samples marinated with lactic acid solution, kefir, and yogurt, whereas yogurt whey‐marinated samples showed almost no change, suggesting a more stable acidification effect in this treatment. The persistence of lower pH values after cooking, particularly in yogurt whey‐marinated samples, suggests that interactions between fermentation‐derived components and muscle proteins may have contributed to maintaining the acidification effect during thermal processing.

The stronger acidifying effect observed in fermented dairy treatments may be related to their compositional complexity. Fermented dairy systems contain residual lactose, peptides, minerals, and microbial metabolites that can enhance acid diffusion and promote interactions with muscle proteins (Augustyńska‐Prejsnar et al. 2021; Sokołowicz et al. 2021). Previous studies on poultry and pork similarly reported that buttermilk, kefir, and acid whey marinades can induce more pronounced physicochemical changes than isolated organic acid solutions, likely due to synergistic effects between fermentation products and milk‐derived bioactive compounds (Simitzis et al. 2021; Zamuz et al. 2019). Moreover, microbial activity and metabolite production in fermented matrices may influence protein structure and ionic balance within muscle tissue more effectively than simple pH‐adjusted acid solutions (Yazgan et al. 2021). The present findings therefore support the view that the structural and biochemical complexity of fermented dairy marinades contributes to pH modulation beyond the effect of lactic acid alone. Similar pH responses have been reported for poultry and red meat marinated with acid whey and fermented dairy‐based marinades (Augustyńska‐Prejsnar, Ormian, Kluz, and Sokołowicz 2019; Sokołowicz et al. 2021).

Marination significantly influenced lightness (L*) values (*p* < 0.05). After marination, all treated samples exhibited higher L* values than the raw control, with yogurt treatment showing the greatest increase. Following heat treatment, L* values increased numerically in the control and yogurt whey‐marinated samples, whereas slight decreases were observed in the remaining treatments; however, none of these changes were statistically significant within treatments (*p* > 0.05). After cooking, yogurt‐marinated meat samples maintained a significantly higher L* value than the control samples (*p* < 0.05), indicating that the observed lightness differences were primarily associated with the marination treatment rather than with thermal processing alone. The enhanced lightness observed particularly in yogurt‐marinated samples may be associated with surface deposition of milk‐derived proteins and accompanying alterations in muscle microstructure, which can increase light scattering and surface reflectance. In addition, heat treatment promotes myofibrillar protein denaturation, contributing to water redistribution within the muscle and further modifying its optical properties (Pathare and Roskilly 2016; Augustyńska‐Prejsnar et al. 2021; Sokołowicz et al. 2021).

Redness (a*) values generally decreased after marination, with the most pronounced reduction observed in yogurt‐treated samples (6.15) compared with the raw control samples (16.49). After cooking, however, redness changes varied among samples depending on the marination treatment. Slight decreases in a* were observed in the control and yogurt whey‐marinated samples, but these were not statistically significant. In contrast, both the lactic acid‐ and yogurt‐marinated samples exhibited significant increases in a* values, while kefir‐marinated samples showed a numerical but nonsignificant increase. Yogurt‐marinated samples nevertheless retained relatively low redness after cooking (9.23), whereas lactic acid‐marinated samples showed the highest a* value (15.36), indicating that pigment stability depended not only on pH but also on the chemical environment created by the marinade matrix. These findings suggest that marinade composition can influence pigment transformation through combined effects on myoglobin structure and redox state, thereby affecting the final color of cooked meat (Suman and Joseph 2013; Wang et al. 2023).

Yellowness (b*) values were also affected by marination. Yogurt whey‐marinated samples exhibited the highest b* value after marination (7.86), while other treatments remained closer to the control. Comparison of marinated and cooked samples showed that b* values increased in all treatments after heating (12.42–16.89), indicating that thermal reactions played a dominant role in yellowness development. However, cooked samples differed significantly in their final b* values. The yogurt‐marinated samples exhibited the lowest yellowness after cooking (12.42), while lactic acid‐, kefir‐marinated samples, and control samples showed higher values, suggesting that marinade composition influenced the extent of thermal color development. Such increases are commonly attributed to nonenzymatic browning processes, particularly Maillard‐type interactions between reducing sugars and amino groups during cooking (Pathare and Roskilly 2016; Suman and Joseph 2013). The comparatively lower yellowness in yogurt‐treated meat may indicate that its matrix limited browning reactions or altered pigment interactions during heating.

Marinade absorption differed significantly among treatments (*p* < 0.05). Yogurt‐marinated samples (12.16%) and lactic acid‐marinated samples (11.54%) showed the highest uptake, kefir‐marinated samples exhibited intermediate absorption (6.30%), and yogurt whey‐marinated samples showed minimal uptake (0.49%). In contrast, the non‐marinated control samples exhibited slight moisture loss during 24 h refrigerated storage, resulting in negative weight change values. Increased uptake is commonly associated with pH‐mediated protein swelling and microstructural pathways that facilitate liquid penetration into muscle fibers (Aktaş et al. 2003). Notably, despite the relatively low marinade absorption values, meat samples marinated with yogurt whey exhibited marked changes in pH and color parameters, indicating that quality modifications were associated not only with absorbed marinade quantity but also with matrix‐dependent interactions between the marinade components and muscle tissue.

Cooking loss values varied numerically among treatments but did not differ significantly (*p* > 0.05). Although yogurt whey‐ (46.23%) and yogurt‐marinated samples (44.62%) showed relatively high losses, kefir‐marinated samples exhibited slightly lower values (40.72%). These tendencies may be linked to matrix‐dependent effects on water migration during heating, including possible contributions of milk‐derived solids and fermentation‐related components present in the marinades, which may form protective layers around muscle fibers (Ismail et al. 2024; Shinde et al. 2018). However, the lack of statistical significance suggests that under the applied conditions, marination treatments influenced pH and color more strongly than cooking yield.

### 3.2 Biogenic Amine Formation

**FIGURE 1:**
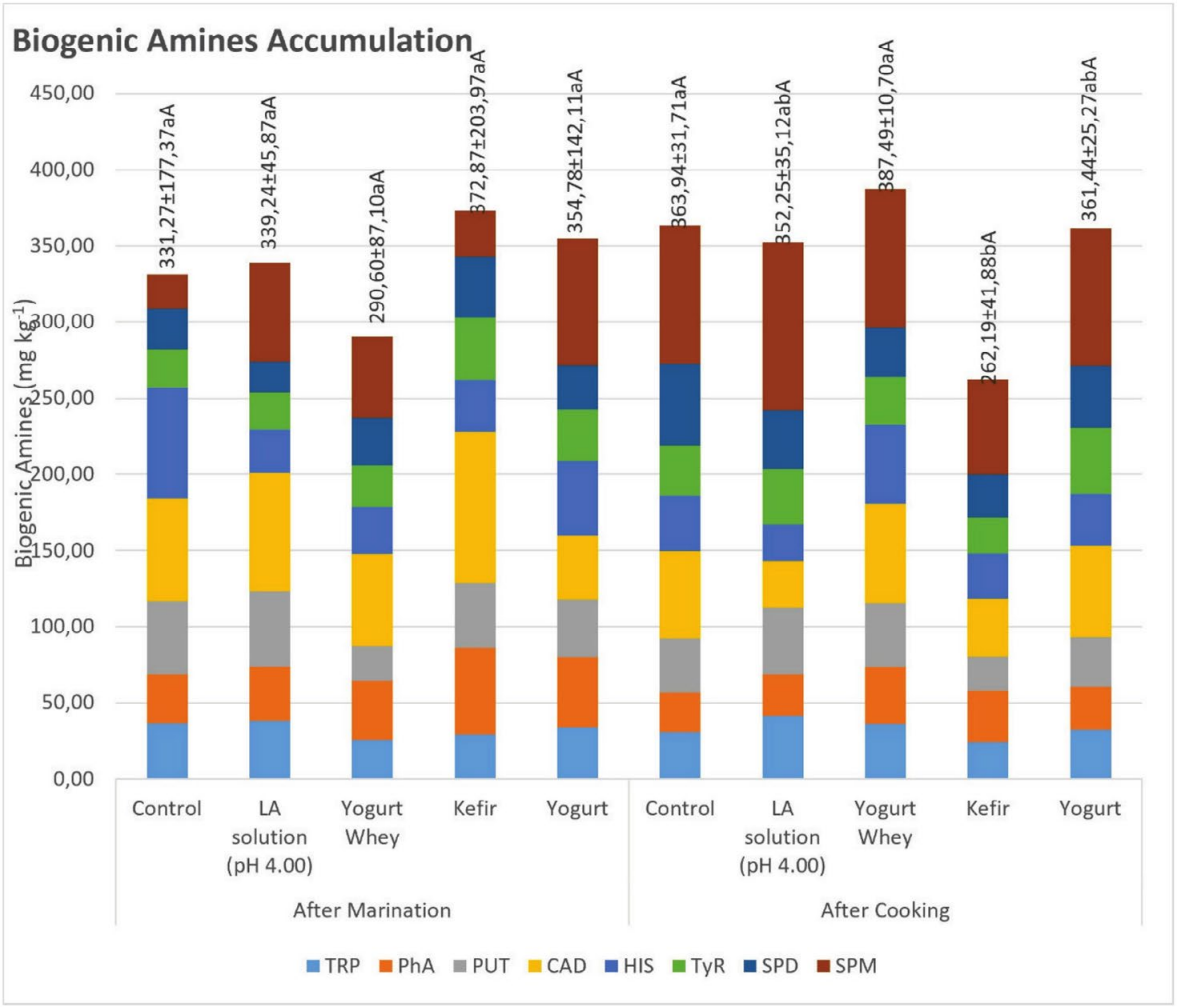
Biogenic amine concentrations (mg kg^−1^) of turkey breast meat subjected to different marination treatments after refrigerated storage (after marination stage) and after cooking. Control samples were stored under the same refrigerated conditions for 24 h without marinade application. Different lowercase letters indicate significant differences among treatments within the same condition (*p* < 0.05). Different uppercase letters indicate significant differences between conditions within the same treatment (*p* < 0.05). Values sharing the same letter are not significantly different (*p* > 0.05). BA, biogenic amines; CAD, cadaverine; HIS, histamine; LA, lactic acid; PhA, 2‐phenylethylamine; PUT, putrescine; SPD, spermidine; SPM, spermine; TRP, tryptamine; TYR, tyramine.

Total biogenic amine (BA) contents of turkey breast meat samples after marination and after cooking are presented in Figure 1. The total BA level of the control samples after marination (331.27 mg kg^−1^) was within the ranges previously reported for poultry meat products. Biogenic amines such as putrescine and cadaverine are commonly associated with microbial activity and product deterioration, whereas histamine and tyramine are more closely related to food safety and toxicological concerns (Gardini et al. 2016; Ruiz‐capillas and Jiménez‐colmenero 2005). After marination, total BA levels increased numerically in most samples; however, no statistically significant differences among samples were detected (*p* > 0.05). Meat samples marinated with yogurt and kefir exhibited higher BA values compared to the control and lactic acid‐marinated samples, whereas yogurt whey‐marinated samples showed a lower value than the control samples. This pattern may be associated with differences in proteolytic activity and the availability of free amino acids that may serve as potential substrates for decarboxylation reactions during refrigerated marination (Doeun et al. 2017; Mozuriene et al. 2016). Nevertheless, all BA levels remained well below concentrations considered hazardous for human consumption.

After cooking, total BA contents showed treatment‐dependent variations rather than a uniform decline, and differences among treatments were not statistically significant (*p* > 0.05). Kefir‐marinated samples tended to show the lowest total BA level after cooking, whereas yogurt whey‐marinated samples and control samples exhibited comparatively higher values. Comparison of marinated and cooked stages further indicated that thermal processing did not consistently reduce total BA levels across treatments, suggesting that the applied heating conditions had only a limited influence on overall amine content. Although thermal processing may contribute to partial degradation of certain amines, the applied cooking conditions did not appear sufficient to markedly reduce total BA levels (Chmiel 2024).

Among individual amines, aliphatic amines such as putrescine and cadaverine were prominent across treatments, reflecting general microbial activity and protein degradation processes typical of fresh meat systems. However, after cooking, polyamines—particularly spermine and, to a lesser extent, spermidine—remained relatively high and in some cases became more pronounced contributors to the total BA profile. This behavior is consistent with their endogenous origin in muscle tissue and their greater thermal stability compared with microbially produced amines (Gardini et al. 2016). In contrast, histamine levels remained low in all samples and well below internationally recognized safety thresholds, indicating that the applied marination treatments did not pose a histamine‐related safety risk (Comas‐Basté et al. 2020).

### 3.3 Sensory Evaluation

**FIGURE 2:**
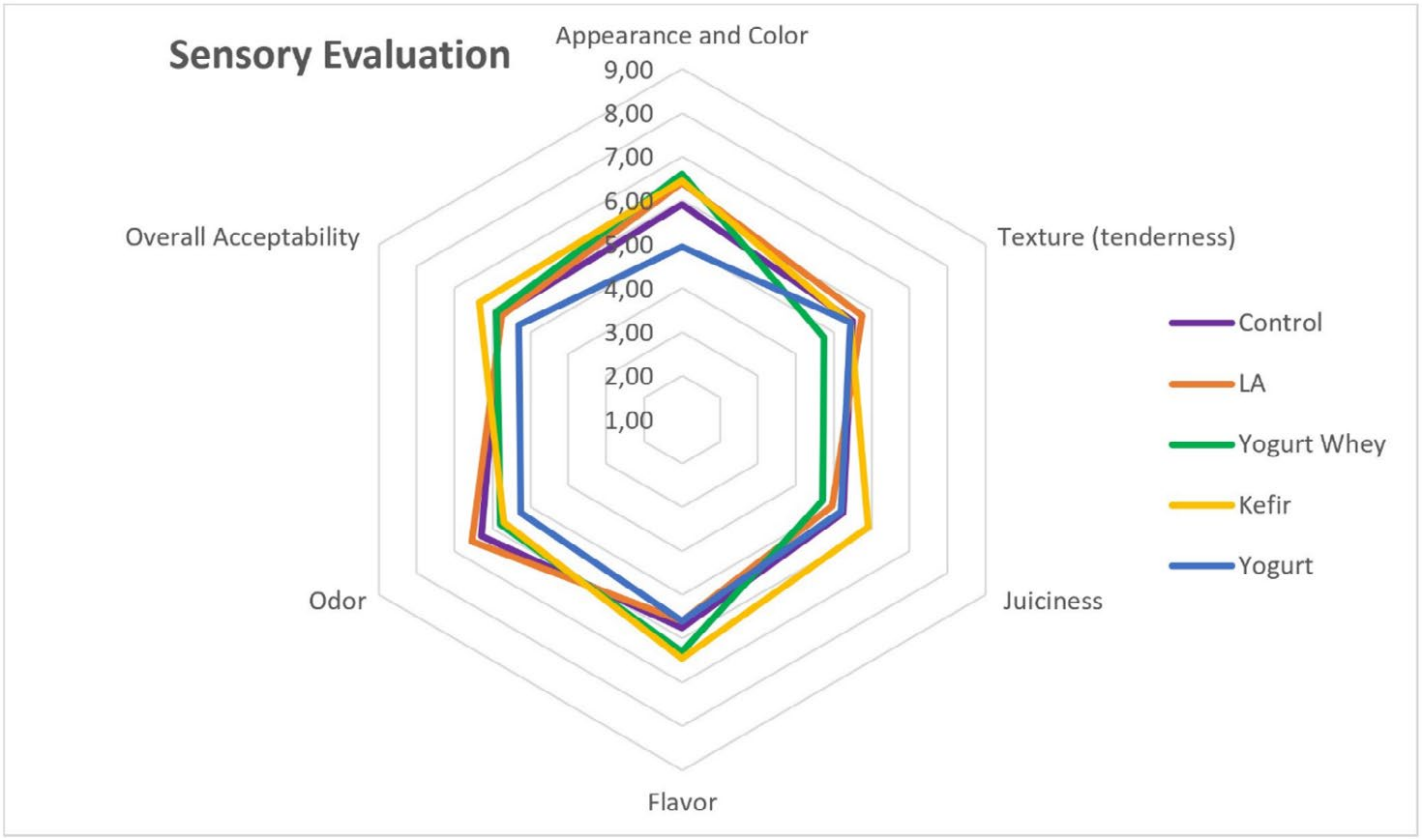
Sensory scores of turkey breast meat marinated with different treatments. Sensory attributes evaluated included appearance and color, texture (tenderness), juiciness, flavor, odor, and overall acceptability using a nine‐point hedonic scale (1 = dislike extremely; 9 = like extremely).

Mean sensory scores for appearance and color, texture (tenderness), juiciness, flavor, odor, and overall acceptability are presented in Figure 2. Significant differences were observed only for appearance/color and odor, whereas the remaining attributes showed comparable scores among treatments.

For appearance and color, yogurt‐marinated samples received significantly lower scores than the other treatments, likely due to excessive surface whitening that reduced visual uniformity. In contrast, yogurt whey‐, kefir‐, and lactic acid‐marinated samples tended to improve appearance scores relative to the control. The lighter surface color and more homogeneous surface appearance observed in meat samples marinated with these treatments may have been positively perceived by panelists, consistent with previous reports on fermented dairy‐based marinades (Ismail et al. 2024).

Texture (tenderness) scores were generally similar across treatments, although lactic acid–marinated samples showed slightly higher numerical values. This tendency may be related to the ability of organic acids to weaken connective tissue and partially solubilize collagen, thereby improving perceived tenderness (Zhu et al. 2020).

Juiciness followed a comparable pattern, with kefir‐marinated samples tending to receive higher scores. This may be associated with their relatively lower cooking loss values and the potential effects of the fermented dairy matrix on water retention and texture stabilization during heating (Gil et al. 2025).

Flavor scores did not differ significantly among treatments; however, yogurt whey‐ and kefir‐marinated samples tended to receive higher scores than the control. Fermented dairy‐based marinades may contribute desirable flavor compounds and volatile metabolites during cooking without imparting excessive acidity (Flores et al. 2019).

For odor, lactic acid‐marinated samples received the highest scores, whereas yogurt‐marinated samples showed lower values. This difference may reflect the perception of a mild acidic aroma as fresh in lactic acid‐marinated samples, while more pronounced sour notes in yogurt‐based marinades may have influenced panel evaluation.

Overall acceptability scores were comparable among treatments, indicating that all marinades produced products with similar consumer acceptance. Nevertheless, kefir‐marinated samples tended to receive the highest numerical scores, suggesting a slight panel preference, although this difference was not statistically significant. This tendency may reflect the combined contribution of appearance, flavor balance, and juiciness to overall liking, a relationship previously reported for fermented dairy‐based marinades (Augustyńska‐Prejsnar et al. 2021; Latoch and Libera 2019).

## 4 Conclusion

This study demonstrated that yogurt whey, kefir, and yogurt marinades produced distinct effects on the physicochemical properties, sensory characteristics, and biogenic amine profile of turkey breast meat. Yogurt whey showed the strongest acidification effect, while kefir provided a balanced sensory and physicochemical profile with comparatively lower biogenic amine values after cooking. Overall, fermented dairy‐based marinades improved quality characteristics without promoting excessive biogenic amine accumulation. Among the tested treatments, yogurt whey showed potential as an alternative marinade system and may contribute to the utilization of dairy by‐products in meat processing applications. The study was limited to physicochemical, sensory, and biogenic amine evaluations. Therefore, future studies including microbiological, compositional, and shelf‐life analyses are needed to better clarify the mechanisms associated with fermented dairy‐based marinades.

## Author Contributions

**Ercan Sarica:** conceptualization, investigation, writing – original draft, methodology, visualization, writing – review and editing, software, formal analysis, data curation. **Gülsüme Biçakci:** conceptualization, investigation, writing – original draft, methodology, visualization, writing – review and editing, software, formal analysis, data curation.

## Ethics Statement

Sensory evaluation was conducted with the voluntary participation of panelists who provided informed consent prior to the study. According to institutional guidelines, formal ethical committee approval was not required for this type of sensory analysis involving minimal risk and no collection of personal or sensitive data.

## Conflicts of Interest

The authors declare no conflicts of interest.

## Data Availability

The data that support the findings of this study are available from the corresponding author upon reasonable request.
